# Trapping the oligomers: new promises in neurosciences

**DOI:** 10.18632/oncotarget.13800

**Published:** 2016-12-05

**Authors:** Thibaut Imberdis, Mike Robitzer, Véronique Perrier

**Affiliations:** ^1^ Université Montpellier, MMDN, Montpellier, EPHE, Paris, France

**Keywords:** oligothiophenes, SDS-resistant oligomers, amorphous aggregates, detection, decontamination, Neuroscience

Prion diseases are fatal neurodegenerative disorders affecting humans and animals. A critical event in prion diseases is the accumulation in the central nervous system of the abnormally folded PrP^Sc^ protein that is the protease-resistant isoform of a normal cellular protein encoded by the host and called PrP^C^. According to the “protein only” hypothesis proposed by Prusiner [1], prion agents are mostly composed of the pathological PrP^Sc^. During prion replication, PrP^Sc^ can trigger the autocatalytic conversion of PrP^C^ into PrP^Sc^ and catalyze its conversion into a misfolded form that becomes pathological. The conversion process involves several PrP^Sc^ intermediates (dimers, trimers, small soluble oligomers and bigger aggregates) that auto-assemble into proto fibrils, which in turns grow into amyloid fibrils [2]. Large fibrils can break into small fragments (called seeds) that will serve to propagate de novo the prion agents [3]. Due to the unique nature of prion agents and their mode of propagation, both PrP^C^ and PrP^Sc^ have been considered as main targets in the development of diagnostic and therapeutic approaches. The majority of screening assays performed in prion-infected cells to identify new anti-prion drugs have focused on reducing the level of PrP^Sc^. However, most of the time, it is difficult to assess which PrP^Sc^ species are targeted by the drug. Indeed, after proteinase K (PK) treatment and in SDS and reducing conditions, all PrP^Sc^ species are completely dissociated, leading to a monomeric band that corresponds to the PrP(27-30) marker. Thus, reduction of the PrP(27-30) marker reflects a global decrease of PrP^Sc^ levels in the cells that is not always correlated with diminished infectivity. Evidences showed that fibrils serve as “reservoirs” to trap small neurotoxic species [4], thus it might be better to fix their amyloid structure rather than to break them. Caution has to be applied when using anti-PrP compounds because the solubilization of large aggregates could indirectly produce seeds that “boost” the prion replication process [3].

A strategy that has not been explored until now, was to search for small molecules that interact with PrP^Sc^ and favor the formation of multimers/oligomers of PrP(27-30) through a cross-linking process [5] (Figure 1). Although it seems totally contradictory to promote the formation of PrP^Sc^ oligomeric species, supposed to be the most infectious species, we hypothesized that small chemical molecules could block small or large PrP^Sc^ species in a pathway that is not competent for prion replication. In a first study, we identified a family of thienyl pyrimidine compounds that favor resistant-SDS PrP^Sc^ (rSDS-PrP^Sc^) oligomers and diminish prion infectivity both in cell lysates and brain homogenates [5]. Thereafter, studies on Alzheimer’s disease showed that small molecules can be powerful tools for the modulation of amyloid formation cascades and demonstrated that acceleration of fibril formation reduces Aβ_42_ toxicity in human neuroblastoma cells and in rat brain slices [6]. Interestingly, oligomer modulators tend to demonstrate that small molecules can redistribute the equilibrium between the various species of the amyloid cascade and these strategies might be useful to identify new compounds for diagnosis or therapeutic purposes.

**Figure 1:**
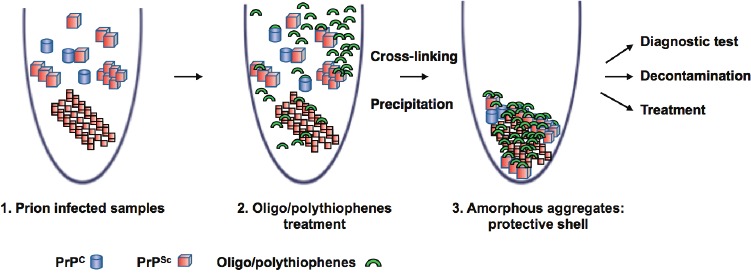
Oligothiophenes trap prions into amorphous aggregates: their potential role in diagnostic, decontamination and therapeutic of neurodegenerative diseases

Due to the low effective doses of the thienyl pyrimidine hits, and on the basis of a structure activity study, we then designed and synthesized a bivalent ligand, called MR100. This compound is effective at nanomolar concentrations in prion-infected cells and has a broad spectrum of action as indicated by its rSDS-PrP^Sc^ oligomer-inducing activity, not only on different rodent strains but also on human prions [7]. Due to strong PrP precipitation property of MR100 (Figure 1), we developed a protocol, called “Rapid Centrifugation Assay” (RCA), based on the ability of MR100 to induce rSDS-PrP^Sc^ oligomers only in prion-infected samples. RCA allows the detection of both PK-sensitive and PK-resistant PrP^Sc^ species in samples of patients with different forms of Creutzfeldt-Jakob disease (CJD). A correlation could be established between the amount of rSDS-PrP^Sc^ oligomers revealed by MR100 and the duration of the symptomatic phase of the disease [7]. This is remarkable because it suggests a link between the amount of the prion biomarker and the duration of the symptomatic phase of the disease in the affected patient, illustrating the potential of MR100 in the diagnosis of prions.

We then asked whether this oligomeric activity induced by MR100 could have an impact on prion infectivity. Pre-incubation of 22L prion-infected brain homogenates with an excess of MR100 before inoculation into mouse brains substantially increased the survival times of animals compared to controls and, remarkably 50% of animals survived without succumbing to the disease [7](Figure 1). This last point means that MR100 has decreased the infectivity of the inoculum by 50%. As MR100 exhibits a strong ability to precipitate PrP isoforms, we hypothesized that an excess of MR100 could form a protective shell around PrP species, which then aggregate and partially inactivate the prion strain, blocking pathways of prion replication (Figure 1). A direct application of this property might be the use of MR100 as a surface prion decontaminant. Interestingly, Herrmann et al., [8] showed that administration of polythiophenes (compound structurally similar to MR100) to the brain of prion-infected mice via osmotic minipumps, led to a survival extension of 80% and demonstrated activity against both mouse and hamster prions [8]. Due to the similarity in chemical structure between MR100 and these polythiophenes, and because polythiophenes have the ability to generate SDS-stable PrP^Sc^ oligomers such as for MR100, we expect that a similar protocol of treatment using MR100 has the potential to delay prion disease in animals.

## References

[1] Prusiner SB. (1982). Science.

[2] Silveira JR (2005). Nature.

[3] Saborio GP. (2001). Nature.

[4] Simoneau S (2007). PLoS Pathog.

[5] Ayrolles-Torro A (2011). J Neurosci.

[6] Bieschke J (2012). Nat Chem Biol.

[7] Imberdis T (2016). Mol Neurodeg.

[8] Herrmann US (2015). Sci transl Med.

